# The Effects of Zinc Treatment on the Blood–Brain Barrier Permeability and Brain Element Levels During Convulsions

**DOI:** 10.1007/s12011-012-9546-y

**Published:** 2012-11-24

**Authors:** Hatice Yorulmaz, Fatma Burcu Şeker, Göksel Demir, İbrahim Ertuğrul Yalçın, Baria Öztaş

**Affiliations:** 1 Halic University School of Nursing, Halic University, Büyükdere Avenue. Number: 101, Mecidiyeköy, 34394 Istanbul, Turkey; 2 Faculty of Medicine, Yeditepe University, Istanbul, Turkey; 3 Engineering Faculty, Environmental Engineering Department, Bahcesehir University, Istanbul, Turkey; 4 Istanbul Faculty of Medicine, Istanbul University, Istanbul, Turkey

**Keywords:** Blood–brain barrier, Convulsions, Elements, Zinc

## Abstract

We evaluated the effect of zinc treatment on the blood–brain barrier (BBB) permeability and the levels of zinc (Zn), natrium (Na), magnesium (Mg), and copper (Cu) in the brain tissue during epileptic seizures. The Wistar albino rats were divided into four groups, each as follows: (1) control group, (2) pentylenetetrazole (PTZ) group: rats treated with PTZ to induce seizures, (3) Zn group: rats treated with ZnCl_2_ added to drinking water for 2 months, and (4) Zn + PTZ group. The brains were divided into left, right hemispheres, and cerebellum + brain stem regions. Evans blue was used as BBB tracer. Element concentrations were analyzed by inductively coupled plasma optical emission spectroscopy. The BBB permeability has been found to be increased in all experimental groups (*p* < 0.05). Zn concentrations in all brain regions in Zn-supplemented groups (*p* < 0.05) showed an increase. BBB permeability and Zn level in cerebellum + brain stem region were significantly high compared to cerebral hemispheres (*p* < 0.05). In all experimental groups, Cu concentration decreased, whereas Na concentrations showed an increase (*p* < 0.05). Mg content in all the brain regions decreased in the Zn group and Zn + PTZ groups compared to other groups (*p* < 0.001). We also found that all elements’ levels showed hemispheric differences in all groups. During convulsions, Zn treatment did not show any protective effect on BBB permeability. Chronic Zn treatment decreased Mg and Cu concentration and increased Na levels in the brain tissue. Our results indicated that Zn treatment showed proconvulsant activity and increased BBB permeability, possibly changing prooxidant/antioxidant balance and neuronal excitability during seizures.

## Introduction

The blood–brain barrier (BBB) protects the brain from the toxic substances in the blood and ensures constant supply of nutrients for neurons. But BBB structure could break down in certain conditions like convulsive seizures [1]. In addition, it was shown that asymmetrical changes in blood brain barrier permeability between hemispheres and cerebellum + brain stem regions during pentylenetetrazol induced seizures in rats [2].

Epileptic seizures strongly modify internal conditions within the nervous tissue. There are many changes in neurotransmitter release, gene activation, and elemental composition [3].

Moreover, it was reported that trace element concentrations like magnesium (Mg), zinc (Zn), copper (Cu), and iron (Fe) concentrations show changes during epileptic seizures. Element contents determine susceptibility to convulsions [4].

BBB is important for Zn homeostasis in the brain. Deficiency or excess of Zn has been shown to contribute to alterations in behavior, abnormal central nervous system development, and neurological disease [5]. Zn is a component of more than 300 different enzymes that function in many aspects of cellular metabolism, involving metabolism of proteins, lipids, and carbohydrates. The brain contains a high concentration of Zn. It serves as a mediator of cell–cell signalling in the central nervous system and alters behavior of channels and receptors [6]. Zn passes into portal blood and is transported. The transport of Zn into the brain parenchyma occurs via the brain barrier system, i.e., the BBB—cerebrospinal fluid barriers. Studies in the hippocampus, amygdala, and neocortex revealed that these neurons in the forebrain represent a subgroup of excitatory glutamatergic neurons called nowadays as gluzinergic neurons [7, 8]. Neurons that contain free Zn ions in the vesicles of their presynaptic boutons are present also in other brain areas and are generally termed Zn-enriched (ZEN) neurons. In the spinal cord, it has been proven that the majority of ZEN terminals are inhibitory g-aminobutyric acid (GABA)-ergic, and only a minor pool is glutaminergic [9]. Glycinergic ZEN neurons in the spinal cord have also been described, and recently, the presence of GABAergic ZEN terminals has been found in the cerebellum [10]. Releasable, vesicular Zn is most abundant in brain regions that are prone to seizures, namely the limbic regions. Potential roles for synaptic released Zn include inhibition of NMDA receptors, potentiation of AMPA receptor responses, inhibition epileptics, in which plasma Mg concentrations decreased of GABA_A_ receptors that lack γ subunits, and antagonism of voltage-gated calcium channels. Thus, as a potential neuromodulator, Zn is capable of exerting effects that could either inhibit or promote neuronal excitability, suggesting the possibility of both pro- and anticonvulsant properties [7, 11].

Therefore, Zn homeostasis in the brain may be important for the development of seizures. In some studies, Zn showed anticonvulsant effect, although Zn has a proconvulsant effect depending on the type of seizure, animal species, and convulsant agents [12, 13]. Systemic charges of Zn have shown anticonvulsant property in several experimental models [12].

Lynes et al. characterized Zn as an agent modifying reactive oxygen species activation, increasing oxidative activity of NADPH, and consequently, triggering oxidative stress [14]. Zn is also cofactors of some antioxidant enzymes such as superoxide dismutase and catalase [15]. It was found that the development of such attacks is related to oxidative stress within the brain In addition, oxidative stress represents one of the crucial factors providing greater BBB permeability values during epileptic seizures [16]. Therefore, antioxidant levels may be important during seizures

The effect of Zn treatment on convulsions is controversial, and its effect on the BBB permeability is not known. Seizure activity in epileptic patients and experimental models of epilepsy influence the metabolism of essential trace elements, e.g., Zn, Fe, Cu, and Mg in the brain and peripheral tissues [5]. It is important to show whether the concentration of essential elements in the brain is altered by enhancement of susceptibility to seizures or epileptic neuronal activity. For these reasons, the aim of the present study was to investigate the role of chronic Zn treatment on the BBB permeability in pentylenetetrazole (PTZ)-induced seizures and levels of other elements in the different brain regions.

## Materials and Methods

### Animals

The experimental procedure was in accordance with the Helsinki Declaration (2004). The 32 Wistar albino rats were divided into four groups as follows: (1) control group, (2) pentylenetetrazole (Sigma Cat no. P6500) group: rats treated with PTZ (80 mg/kg, i.v.) to induce epileptic seizures, (3) Zn group: rats treated with ZnCl_2_ 227 ml/kg [17] added to drinking water for 2 months, and (4) Zn + PTZ group. Cannulations of the femoral artery and vein were performed in animals. The blood pressure was measured from the femoral artery. PTZ and Evans blue were injected from the femoral vein. Because most anesthetics have anticonvulsant properties, diethyl ether anesthesia was used.

### Assessment of Blood–Brain Barrier Permeability

Evans Blue (Sigma Cat no. E-2129) was used as BBB tracer (4 ml/kg, i.v.). PTZ was i.v. injected in order to induce convulsions, 5 min after Evans Blue injection. The brains were elutriated by perfusion with 0.9 % NaCl solution via the left ventricle 25 min after Evans Blue injection. Then, the brains were dissected and separated into the left hemisphere, right hemisphere, and cerebellum + brainstem regions. Wet masses of the dissected samples were measured. Afterwards, each brain region was homogenized by placing in tubes with phosphate buffer (2.5 ml). A 60 % solution of trichloroacetic acid (2.5 ml) was put in each tube after homogenizing; this procedure provided separation of Evans Blue from albumin by vortexing for 2 min. After the vortex process, the tubes were kept at 4 °C for 30 min and centrifuged at 1,000×*g* at 4 °C for 30 min. After centrifuging, the absorbance values were read spectrophotometrically at 620 nm wavelength (after decanting supernatants to spectrophotometer tubes). The Evans Blue content values (micrograms per milligram tissue) were calculated for brain tissue samples using the Evans Blue absorbance-quantity regression equation and catenary via the obtained absorbance values. The statistical analysis was made with SPSS (Statistical Package for Social Sciences 16.0 version).

### Measurement of Element Concentrations

Samples of 0.2 g. were taken and transferred into Teflon vessels and then 10 ml of 20 % HNO3 (Merck) was added. Samples were mineralized in a microwave oven (Berghof–MWS2) as follows: at 145 °C for 5 min, at 165 °C for 5 min, and at 175 °C for 20 min. After cooling, the samples were filtered by Whatman filters, and filled up to 50 ml with ultrapure water in volumetric flasks and then stored in falcon tubes. Standard solutions were prepared by using multi-element stock solutions—1,000 ppm (Merck), and element (Cu, Mg, Na, and Zn) measurements were done by inductively coupled plasma optical emission spectroscopy (PerkinElmer-Optima 7000 DV).

### Statistical Analysis

The obtained data were analyzed with SPSS (version 17.0) statistics program. Evans Blue–albumin extravasation, arterial pressure, and element concentration were expressed as mean ± standard deviation. Data were compared among groups and brain regions using one-way analysis of variance and subsequently Tukey’s test was performed (*P* < 0.05).

## Results

Systolic and diastolic blood pressures are shown in Table 1. PTZ-induced convulsions caused a significant increase in the blood pressure in groups 2 and 4 (*P* < 0.001).

**Table 1:** Before and after PTZ, values of arterial blood pressure for experimental groups

Groups	*n*	Diastolic pressure (mmHg)	Systolic pressure (mmHg)	Diastolic pressure (mmHg)	Systolic pressure (mmHg)
		Before PTZ	Before PTZ	After PTZ	After PTZ
Control	8	80 ± 3	100 ± 5	−	−
PTZ	8	72 ± 2	92 ± 6	148 ± 9	170 ± 10
Zn	8	76 ± 5	96 ± 8	−	−
Zn + PTZ	8	88 ± 4	108 ± 3	142 ± 9	152 ± 12

*P < 0.001, significant difference from control group

**Fig. 1:**
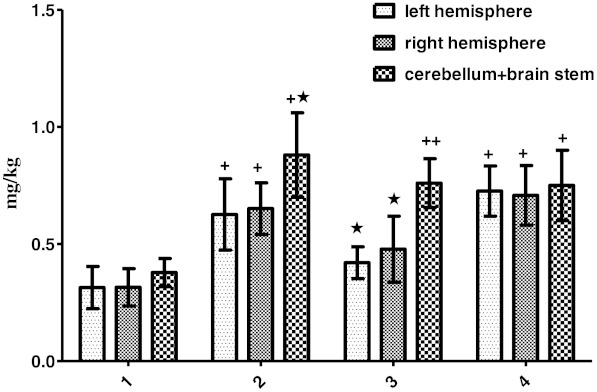
Displaying Evans Blue (EB) amounts in the left and right hemispheres and cerebellum + brain stem of the groups and within groups (control group *1*, PTZ group *2*, Zn group *3*, Zn + PTZ group *4*). One *star* shows a significant difference (*P* < 0.05). One *cross* shows a significant difference (*P* < 0.01). Control vs. other groups and the region of the brain within groups

In control animals (group 1), the mean Evans Blue concentrations in both cerebral hemispheres and in the cerebellum + brainstem regions were 0.31 ± 0.09, 0.31 ± 0.08, 0.37 ± 0.07 (mean ± SD) μg/mg tissue, respectively. The BBB permeability increased significantly in the PTZ group [0.62 ± 0.15, 0.65 ± 0.11, 0.88 ± 0.18 (mean ± SD) μg/mg tissue, respectively]. The mean Evans Blue concentration in the Zn group is [0.42 ± 0.06, 0.47 ± 0.14, 0.76 ± 0.10 (mean ± SD) μg/mg tissue, respectively] and [0.72 ± 0.11, 0.70 ± 0.13, 0.75 ± 0.16 (mean ± SD) μg/mg tissue, respectively] in the Zn + PTZ groups (*P* < 0.05 and *P* < 0.01; Fig. 1).

There was no significant difference between the control and Zn + PTZ groups (*P* > 0.05). In both the Zn and PTZ groups, the mean Evans Blue concentration of cerebellum + brainstem increased compared to cerebral hemispheres (*P* <0.05, *P* < 0.01, and *P* < 0.001; Fig. 1).

**Fig. 2:**
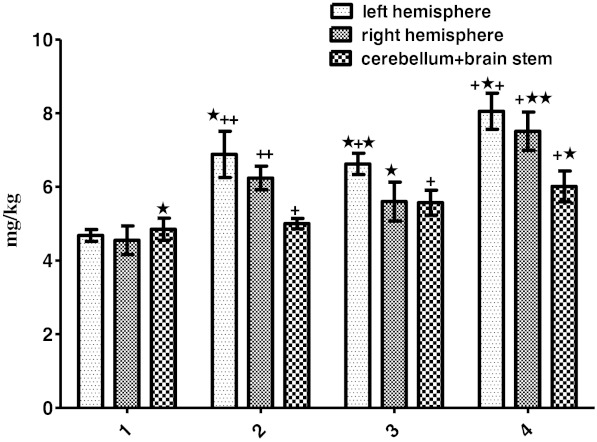
Amounts of Zn (milligrams per kilogram tissue) in samples of the left brain hemisphere, right hemisphere, and cerebellum + brainstem regions in groups *1–4* of experimental rats and within groups (control group *1*, PTZ group *2*, Zn group *3*, Zn + PTZ group *4*). Intragroup means ± SD are shown. One *cross* shows a significant difference (*P* < 0.01). One *star* shows a significant difference (*P* < 0.05). Control vs experimental groups. Zn + PTZ group vs Zn and PTZ group and the region of the brain within groups

Zn concentrations were significantly increased in all brain regions and in all groups compared to control group (*P* < 0.01 and *P* < 0.05). Additionally, it increased in all the brain regions of the Zn + PTZ group compared to PTZ and Zn groups (*P* < 0.05; Fig. 2).

Zn concentration of cerebellum + brainstem significantly increased when compared to right hemisphere in the control group (*P* < 0.05). It was significantly high in brain hemispheres when compared to cerebellum + brainstem (*P* < 0.01), and also, Zn concentration of the left hemisphere was significantly higher than the right hemisphere (*P* < 0.05).

Zn concentration of the left hemisphere in Zn group was higher than the cerebellum + brainstem and right hemisphere (*P* < 0.05 and *P* < 0.01). In the Zn and PTZ groups, Zn concentration of the left and right hemispheres increased compared to cerebellum + brainstem regions (*P* < 0.05 and *P* < 0.01; Fig. 2).

**Fig. 3:**
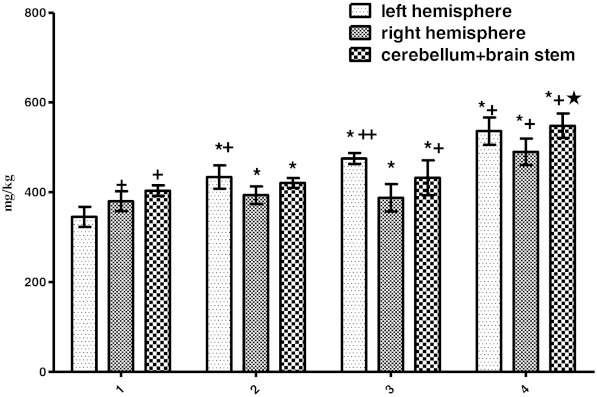
Amounts of Na (milligrams per kilogram tissue) in samples of the left brain hemisphere, right hemisphere, and cerebellum + brainstem regions in groups *1–4* of experimental rats and within groups (control group *1*, PTZ group *2*, Zn group *3*, Zn + PTZ group *4*). Intragroup means ± SD are shown. One *asterisk* shows cases of significant differences (*P* < 0.001) One *cross* shows a significant difference (*P* < 0.01). Control vs experimental groups. Zn + PTZ group vs Zn and PTZ group and the region of the brain within groups

Significant increases of Na content in all groups were observed (*P* < 0.001; Fig. 3). In the control group, Na level in cerebellum + brainstem was higher than the left hemisphere (*P* < 0.01) and significantly increased in the left hemisphere compared to the right hemisphere (*P* < 0.01). In the PTZ and Zn group, Na concentration of the left hemisphere increased compared to the right hemisphere (*P* < 0.01). In the Zn group, Na concentration of the left hemisphere increased compared to the cerebellum + brainstem (*P* < 0.05). In the Zn and Zn + PTZ groups, Na concentration of cerebellum + brainstem region increased compared to the right hemisphere (*P* < 0.05; Fig. 3).

**Fig. 4:**
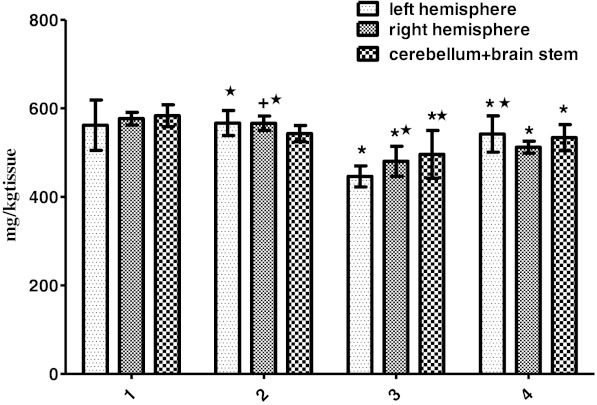
Amounts of Mg (milligrams per kilogram tissue) in samples of the left brain hemisphere, right hemisphere, and cerebellum + brainstem regions in groups *1–4* of experimental rats (control group *1*, PTZ group *2*, Zn group *3*, Zn + PTZ group *4*) and within groups. Intragroup means ± SD are shown. One *asterisk* shows cases of significant differences (*P* < 0.001) Control vs experimental groups and the region of the brain within groups

A significant reduction of Mg content in all the brain regions was observed in Zn-treated groups compared to other groups (*P* < 0.001; Fig. 4). Mg level was found high in the cerebellum + brain stem when compared to cerebral hemispheres and found high in the right hemisphere in the PTZ group (*P* < 0.05 and *P* < 0.01).

**Fig. 5:**
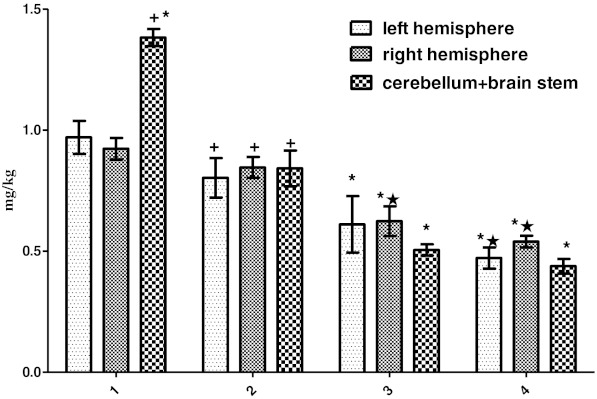
Cu (milligrams per kilogram tissue) in samples of the left brain hemisphere, right hemisphere, and cerebellum + brainstem regions in groups *1–4* of experimental rats (control group *1*, PTZ group *2*, Zn group *3*, Zn + PTZ group *4*) and within groups. Intragroup means ± SD are shown. One *asterisk* shows cases of significant differences (*P* < 0.001) One *cross* shows a significant difference (*P* < 0.01). Control vs experimental groups and the region of the brain within groups

In the Zn-treated group, Mg level in the right hemisphere increased compared to the left hemisphere and the cerebellum + brainstem (*P* < 0.05 and *P* < 0.01). In the Zn + PTZ group, Mg concentration of the left hemisphere was higher than the right hemisphere (*P* < 0.05; Fig. 4). Cu content in all the brain regions was significantly decreased in all groups compared to the control (*P* < 0.01 and *P* < 0.001; Fig. 5).

In the control group, Cu concentration of cerebellum + brainstem increased compared to cerebral hemispheres (*P* < 0.01 and *P* < 0.001). In the Zn group, Cu concentration of the right hemisphere increased compared to the cerebellum + brainstem region (*P* < 0.05 and *P* < 0.01). Cu concentration of the left and right hemispheres increased compared to the cerebellum + brainstem regions in Zn + PTZ group (*P* < 0.05).

## Discussion

In our study, we observed that BBB permeability significantly increased in the PTZ group as shown in numerous studies [18]. We found that BBB breakdown is significantly higher than cerebral hemispheres. Arnaiz et al. [19] showed that the cerebellum is more susceptible to epileptic seizures, and its antioxidant capacity is lower than cerebral cortex, and brain imaging studies in human have shown that cerebral metabolic rate of glucose consumption is high in the occipital cortex and cerebellum The increased metabolism caused by high firing rates of the cerebellum neurons may provoke more free radical production during epileptic seizures. These factors can be a reason for high Evans Blue values in comparing to brain hemispheres in the PTZ group [19].

Also, chronic Zn supplementation for 2 months increased BBB permeability during convulsions. These results demonstrated that BBB was severely damaged in both Zn-supplemented and not supplemented epileptic animals. Several reports suggested that the body electrolytes Na^+^, K^+^, Ca^2+^, and Mg^2+^ and the level of some trace elements play a vital role for seizure conditions to develop [20, 21]. It is known that Zn is a vital nutrient, and with the exception of iron, it is the most abundant trace element in the body. Zn released from neuron terminals serves a neuromodulatory function, as suggested by the multiple selective actions of Zn on synaptic function and the potency of Zn ions to modulate numerous neurotransmitter receptors such as GABA, NMDA, and ion channels [5, 9, 22]. As a potential neuromodulator, Zn is capable of exerting effects that could either inhibit or promote neuronal excitability, suggesting the possibility of both pro- and anti-convulsant effects [23, 24]. In this study, Zn supplementation for 2 months did not show a protective effect on increased BBB permeability during convulsions. Moreover, there was no change in severity and duration of seizure intensity and arterial blood pressure between animals treated with Zn and those that were not treated with Zn. In parallel with our results, mice that are lacking vesicular Zn (pharmacologically or genetically) or rats that are fed a Zn-deficient diet have been shown to be more susceptible to kainate-induced seizures and show an increase in seizure severity and duration [25]. Conversely, intracerebral or intrahippocampal injections of Zn have been shown to cause partial and secondarily generalized seizures in rabbits [26, 27]. Taken together, these data indicate that alterations of Zn homeostasis contribute to the development of epileptic seizures. These contradictions are probably related to the fact that Zn manifests dissimilar effects depending on the type of application form, doses, and duration.

We found that Zn by itself also caused BBB breakdown without seizures. We think that Zn might cause an imbalance between ionic homeostasis in the brain. Significant increases of Zn amount in all brain regions under study were observed in all groups compared to the control group. Moreover, Zn concentration significantly increased in the Zn + PTZ group compared to PTZ and Zn groups. Studies in hippocampus, amygdale, and neocortex showed that the neurons in these regions represent a group of excitatory gluzinergic neurons neurons It has been shown that most of the Zn in these Zn-enriched neurons are found in their presynaptic boutons [7, 8]. In addition to Zn released from neuron terminals, chronic Zn treatment may cause Zn accumulation. In our results, there is a correlation between Zn levels and Evans blue values in PTZ and Zn + PTZ groups. According to this result, Zn accumulation may provoke BBB breakdown during epileptic seizures. Zn is a potent Na-K-ATPase inhibitor. Barbeau et al. showed that intracerebroventricular Zn inhibited Na-K-ATPase and resulted in convulsions by increasing cerebrospinal fluid potassium levels [27]. Inhibiting Na-K-ATPase in cerebral endothelial cells may be a significant reason for BBB damage.

On the other hand, we found a significant decrease in Cu levels in all experimental groups. Cu is an essential trace element in all living organisms. It is mainly a part of the active center of cuproenzymes, such as cytochrome c oxidase—a component of the mitochondrial respiratory chain [28]. One of the symptoms of Cu deficiency includes decreased superoxide dismutase, ceruloplasmin as well as cytochrome-c oxidase [29]. During epileptic seizures, free radical production is increased and insufficiency in antioxidant defence mechanisms could provoke BBB breakdown and neuronal damage. Sahin et al. [30] have shown decreased levels of Cu and Fe in brain tissue and increase in BBB permeability in the repeated seizures group. It has been shown that taking large doses of supplemental Zn over extended periods of time has shown to be associated with Cu deficiency [31]. These findings are parallel with our results. When we consider that free radical levels are increased in epileptic seizures, the antioxidant defense system in epileptic animals might remain insufficient in these groups because of Cu deficiency.

We found a significant increase in the amount of Na in the three brain regions of all the groups. Also, it was found that Na level was increased in the Zn + PTZ group compared to the PTZ and Zn groups. It was shown that brain shrinkage induced by hypernatremia can cause rupture of cerebral veins, with focal intracerebral and subarachnoid hemorrhages, which in turn can provoke seizures [32]. Seizures also occur in ≤40 % of patients treated for severe hypernatremia by rapid infusion of hypotonic solutions [33]. In our study, Mg^2+^ was unaltered in all brain regions in the PTZ group, and this is consistent with many studies [34]. Mg^2+^ is essential in neuronal excitability. It alters Ca^2+^ mobilization and stabilizes excitable membranes and also exerts a voltage-dependent blockage of the NMDA-receptor channel [35]. In human beings hypomagnesemia is associated with manifestations of central nervous system dysfunction [36]. Mg^2+^ levels in all the brain regions were decreased in the Zn group and Zn + PTZ groups compared to other groups. Decreased Mg^2+^ level may contribute to BBB damage.

During convulsions, Zn treatment did not show a protective effect on the BBB permeability. Also, we have shown that Zn has adverse effects on the BBB via changing Mg, Cu, Na, and Zn levels. Chronic Zn treatment by itself decreased Mg and Cu concentration and increased Na levels in brain tissue and during seizures and non-seizures. As a result of that, changes in prooxidant/antioxidant balance and neuronal membrane excitability may contribute to BBB damage. Our study showed that Zn treatment showed proconvulsant activity during seizures.

There is a neurochemical asymmetry as well as functional asymmetry in the brain [37]. It may be the underlying reason for difference in element levels in the control group. However, there are more fluctuations in element levels in the experimental groups. Brain hemispheres manifest different neurochemical changes against either extra supplementation of an element such as Zn or a pathological condition such as epilepsy. These neurochemical changes also affect element levels important for electrical activity of the brain. The cause of these differences can be revealed more precisely by molecular studies.

## References

[1] Persidsky Y, Ramirez SH, Haorah J, Kanmogne G (2006). Blood–brain barrier: structural components and function under physiologic and pathologic conditions. J Neuroimmune Pharmacol.

[2] Yorulmaz H, Seker FB, Oztas B (2011) Effect of vitamin E on blood–brain barrier permeability in aged rats with PTZ-ınduced convulsions. Neurophysiology 349–353. doi:10.1007/s11062-011-9168-6

[3] Hamed SA, Abdellah MM (2004). Blood levels of trace elements, electrolytes, and oxidative stress/antioxidant systems in epileptic patients. J Pharmacol Sci.

[4] Carl GF, Critchfield JW, Thompson JL, McGinnis LS, Wheeler GA, Gallagher BB, Holmes GL, Hurley LS, Keen CL (1989). Effect of kainate-induced seizures on tissue trace element concentrations in the rat. Neuroscience.

[5] Takeda A, Tamano H, Ogawa T, Takada S, Ando M, Oku N, Watanabe M (2012). Significance of serum glucocorticoid and chelatable Zn in depression and cognition in zinc deficiency. Behav Brain Res.

[6] Parkin G (2004). Chemistry. Zinc–zinc bonds: a new frontier. Science.

[7] Slomianka L (1992). Neurons of origin of zinc-containing pathways and the distribution of zinc-containing boutons in the hippocampal region of the rat. Neuroscience.

[8] Frederickson CJ, Moncrieff DW (1994). Zinc-containing neurons. Biol Signals.

[9] Danscher G, Jo SM, Varea E, Wang Z, Cole TB, Schrøder HD (2001). Inhibitory zinc-enriched terminals in mouse spinal cord. Neuroscience.

[10] Birinyi A, Parker D, Antal M, Shupliakov O (2001). Zinc co-localizes with GABA and glycine in synapses in the lamprey spinal cord. J Comp Neurol.

[11] Christine CW, Choi DW (1990). Effect of zinc on NMDA receptor mediated channel currents in cortical neurons. J Neurosci.

[12] Sterman MB, Shouse MN, Fairchild MD, Belsito O (1986). Kindled seizure induction alters and is altered by zinc absorption. Brain Res.

[13] Itoh M, Ebadi M (1982). The selective inhibition of hippocampal glutamic acid decarboxylase in zinc-induced epileptic seizures. Neurochem Res.

[14] Lynes MA, Kang YJ, Sensi SL, Perdrizet GA, Hightower LE (2007). Heavy metal ions in normal physiology, toxic stress, and cytoprotection. Ann N Y Acad Sci.

[15] Naziroglu M (2007). New molecular mechanisms on the activation of TRPM2 channels by oxidative stress and ADP-ribose. Neurochem Res.

[16] Oztas B, Akgul S, Seker FB (2007). Gender difference in the influence of antioxidants on the blood–brain barrier permeability during pentylenetetrazole-induced seizures in hyperthermic rat pups. Biol Trace Elem Res.

[17] Dani V, Malhotra A, Dhawan D (2007). 131I induced hematological alterations in rat blood: protection by zinc. Biol Trace Elem Res.

[18] Oztas B, Kaya M (2003). Blood–brain barrier permeability during acute and chronic electroconvulsive seizures. Pharmacol Res.

[19] Arnaiz L, Lores S, Travacio M, Llesuy ZS, Rodriguez de G (1998). Regional vulnerability to oxidative stress in a model of experimental epilepsy. Neurochem Res.

[20] Sheth DP (1997). Hypocalcemic seizures in neonates. Am J Emerg Med.

[21] Ilhan A, Uz E, Kali S, Var A, Akyol O (1999). Serum and hair trace elements levels in patients with epilepsy and healthy subjects: does the antiepileptic therapy affect the element concentrations of hair. Eur J Neurol.

[22] Hollman M, Boulter J, Maron C, Beasley L, Sullivan J, Pecht G, Heinemann S (1993). Zinc potentiates agonist-induced currents at certain splice variants of the NMDA receptor. Neuron.

[23] Harrison NL, Gibbons SJ (1994). Zn^+2^: an endogenous modulator of ligand and voltage-gated ion channels. Neuropharmacol.

[24] Fukahori M, Itoh M, Oomagari K, Kawasaki H (1988). Zinc content in discrete hippocampal and amygdaloid areas of the epilepsy (El) mouse and normal mice. Brain Res.

[25] Takeda A, Hirate M, Tamano H, Nisibaba D, Oku N (2003). Susceptibility to kainate-induced seizures under dietary zinc deficiency. J Neurochem.

[26] Pei Y, Zhao D, Huang J, Cao L (1983). Zinc-induced seizures: a new experimental model of epilepsy. Epilepsia.

[27] Barbeau A, Donaldson J (1974). Zinc, taurine and epilepsy. Arch Neurol.

[28] Puig S, Thiele DJ (2002). Molecular mechanisms of copper uptake and distribution. Curr Opin Chem Biol.

[29] Sandstead HH (1995). Requirements and toxicity of essential trace elements, illustrated by zinc and copper. Am J Clin Nutr.

[30] Sahin D, Ilbay G, Ates N (2003). Changes in the blood–brain barrier permeability and in the brain tissue trace element concentrations after single and repeated pentylenetetrazole-induced seizures in rats. Pharmacol Res.

[31] Ogiso T, Moriyama K, Sasaki S, Ishimura Y, Minato A (1974). Inhibitory effect of high dietary zinc on copper absorption in rats. Chem Pharm Bull (Tokyo).

[32] Adrogue HJ, Madias NE (2000). Hypernatremia. New Eng J Med.

[33] Reeves WB, Bichet DG, Andreoli TE, Wilson JD, Foster DW, Kronenberg HM, Larsen PR (1998). William’s textbook of endocrinology. Posterior pituitary and water metabolism, 9th edn..

[34] Kürekçi AE, Alpay F, Tanindi S, Gokcay E, Ozcan O, Akin R (1995). Plasma trace element, plasma glutathione peroxidase, and superoxide dismutase levels in epileptic children receiving AEDs therapy. Epilepsia.

[35] Agus Z, Wassersteın A, Goldfarb S (1982). Disorders of calcium and magnesium homeostasis. Am J Med.

[36] Czeh G, Somjen GG (1989). Changes in extracellular calcium and magnesium and synaptic transmission in isolated mouse spinal cord. Brain Res.

[37] Glick SD, Shapiro KM (1984) Functional and neurochemical asymmetries. In: N. Geschwind A.M. Galaburda (eds) Cerebral dominance. Academic Press, New York, pp 147–166

